# Phenomic Studies on Diseases: Potential and Challenges

**DOI:** 10.1007/s43657-022-00089-4

**Published:** 2023-01-05

**Authors:** Weihai Ying

**Affiliations:** 1 Med-X Research Institute and School of Biomedical Engineering, Shanghai Jiao Tong University, 1954 Huashan Road, Shanghai, 200030 China; 2 Collaborative Innovation Center for Genetics and Development, Shanghai, 200043 China

**Keywords:** Phenomics, Diseases, Big data, Precision medicine, Diagnosis

## Abstract

The rapid development of such research field as multi-omics and artificial intelligence (AI) has made it possible to acquire and analyze the multi-dimensional big data of human phenomes. Increasing evidence has indicated that phenomics can provide a revolutionary strategy and approach for discovering new risk factors, diagnostic biomarkers and precision therapies of diseases, which holds profound advantages over conventional approaches for realizing precision medicine: first, the big data of patients' phenomes can provide remarkably richer information than that of the genomes; second, phenomic studies on diseases may expose the correlations among cross-scale and multi-dimensional phenomic parameters as well as the mechanisms underlying the correlations; and third, phenomics-based studies are big data-driven studies, which can significantly enhance the possibility and efficiency for generating novel discoveries. However, phenomic studies on human diseases are still in early developmental stage, which are facing multiple major challenges and tasks: first, there is significant deficiency in analytical and modeling approaches for analyzing the multi-dimensional data of human phenomes; second, it is crucial to establish universal standards for acquirement and management of phenomic data of patients; third, new methods and devices for acquirement of phenomic data of patients under clinical settings should be developed; fourth, it is of significance to establish the regulatory and ethical guidelines for phenomic studies on diseases; and fifth, it is important to develop effective international cooperation. It is expected that phenomic studies on diseases would profoundly and comprehensively enhance our capacity in prevention, diagnosis and treatment of diseases.

## Introduction

The societies in many countries around the world are undergoing rapid acceleration of aging. As reported by the seventh national census of China in 2020, there were up to 264 million people ages 60 and older, accounting for 18.7% of the total population of China, while there were up to 190 million people ages 65 and older, accounting for 13.5% of the total population of China (Tu et al. 2022). Since aging is a key risk factor for a number of major diseases, the aging of the society is leading to significant increases in the patients of age-related diseases (Campisi et al. 2019; Hou et al. 2019; North and Sinclair 2012; Partridge et al. 2018). In 2019, there were 3.94 million new stroke cases in China, with the incidence rate of stroke increasing by 86% from 1990 to 2019; and there were 2.19 million stroke-caused deaths in China in 2019, with the mortality rate increasing by 32.3% from 1990 to 2019 (Ma et al. 2021; Wang et al. 2022). The causes of these severe conditions are multifactorial, including limitations of effective drugs (Malik et al. 2021; Peña et al. 2017) and significant deficiency in medical imaging equipment in many areas, particularly in rural areas (Hricak et al. 2021; Khatib et al. 2018). To overcome these severe challenges, novel preventive strategies, diagnostic approaches and therapeutic strategies are urgently and critically needed. Increasing evidence has suggested that phenomics has provided a revolutionary strategy and approach for discovering new risk factors (Andrews et al. 2021; Nikpay and Mohammadzadeh 2020; Went et al. 2020; Yang et al. 2022; You et al. 2022), diagnostic biomarkers (Nicholson 2021; Xu et al. 2022; Zhang et al. 2022a) and precision therapies (Delude 2015; Nicholson 2021; Robinson 2012; Zhang et al. 2021) for human diseases.

The term ‘phenomics’ was coined by Dr. Steven A. Garan in his speech in 1996 (Jin 2021). Phenomics describes the measurement of the phenome, which is a series of measurable traits including physical, chemical, and biological traits of individuals and populations. The traits result from complex interactions among genetic factors, environmental factors, diet and symbiotic microorganisms (Jin 2021).

The data from phenomic studies are not merely regular big data of biomedicine, or an accumulation of biomedical data using various biomedical technologies. Compared with conventional studies on diseases, phenomics-based studies on diseases have the following distinct properties: first, standardization in all aspects of the studies, including the methods of measurements, data storage, data distribution and data analyses, which are required by artificial intelligence (AI)-based analyses; second, big data-driven research; and third, a majority of the data from phenomic studies are massive, multi-dimensional and structured, while such non-structured information as medical images and questionnaire also plays critical roles in phenomic studies. It is noteworthy that the data from phenomic studies on diseases belong to critical big data of biomedicine, which have the distinct characteristics as mentioned above.

After the completion of the Human Genome Project, it has become increasingly crucial to conduct comprehensive studies on human phenome, which may provide a fundamental basis for deciphering the codes for human health. International scientific community has recognized that phenomic studies can lead to elucidation of the mechanisms underlying the relationships among phenome, genome and environmental impact. An article published in *Nature Review Genetics* stated that there is significant deficiency in not only the information about phenome, but also the capability of studying phenome, which indicates urgent need for phenomic studies (Houle et al. 2010). It is reasonable to expect that phenomic studies, like genomic studies, would provide a revolutionary driving force for the development of new strategies and methods for biomedicine.

Phenomics has been established on the basis of the following development: first, there has been rapid development of multi-omics technologies that include genomics, proteomics, metabolomics, radiomics, epigenomics, transcriptomics and microbiomics (Hasin et al. 2017; Leon-Mimila et al. 2019; Olivier et al. 2019; Panunzio et al. 2021). Development of numerous new technologies including multi-omics technologies has enabled us to conduct large scale, multi-dimensional determinations on the biological properties of human subjects; second, development of AI and big data science has made it possible to analyze the big data from phenomic studies (Biswas and Chakrabarti 2020); third, development of modern biology and medicine has exposed significant relationships among various biological properties of human, indicating the potential and importance of not only determining the properties of individual units of life, including individual organs, tissues, cells and molecules, but also investigating the relationships among the biological properties of these units; fourth, it is required by precision medicine that multi-dimensional data of patients are collected and analyzed (Delude 2015; Houle et al. 2010; Newgard and Attie 2010; Nicholson 2021; Zhang et al. 2021); and fifth, cumulative evidence has indicated that the information from genetic studies on patients is far from sufficient for elucidating the mechanisms of diseases and establishing novel strategies for diagnosis and treatment of diseases (Delude 2015; Hasin et al. 2017; Wu et al. 2016).

Increasing evidence has indicated the significance and urgency for applications of phenomics in studying diseases (Delude 2015; Houle et al. 2010; Newgard and Attie 2010; Nicholson 2021; Zhang et al. 2021). However, since it is still in early developmental stage, phenomic studies on disease are facing multiple major challenges and tasks. The major aim of this article is to provide an overview of the significance and potential of phenomic studies on diseases, the current status of the research field, as well as the major challenges and tasks facing the research field.

## Significance of Phenomic Studies on Diseases

With the increase in the life span of the human populations, there have been significant increases in the incidence of age-associated diseases (Hou et al. 2019; North and Sinclair 2012; Partridge et al. 2018). While there has been remarkable progress in our understanding on major diseases, cumulating evidence has indicated urgency to apply novel strategies to discover new risk factors, and to establish new diagnostic approaches and therapeutic strategies for diseases. For example, there has been only one drug approved by US Food and Drug Administration (FDA) for treating ischemic stroke—Recombinant Tissue-Type Plasminogen Activator (r-tPA), which was approved by FDA in 1996 (Marko et al. 2020; Zivin 2009). Due to its very limited therapeutic window and toxic side effects, only a small percentage of acute ischemic stroke (AIS) patients can be treated by the drug (Peña et al. 2017). During the last three decades, a number of clinical trials on the drugs for treating stroke have failed (Kaur et al. 2013; Matur et al. 2022). These pieces of information have indicated that human being is still far from conquering major diseases, which also indicates the critical significance of applying novel and revolutionary approaches to prevent, diagnose and treat diseases.

Increasing evidence has also indicated that it is insufficient to realize precision medicine by conducting solely genetic studies on major diseases: first, environmental factors may play crucial roles in the pathogenesis of numerous diseases, e.g., an article published in *Nature* reported that external risk factors play key roles in carcinogenesis, while internal risk factors contributed only 10–30% risk of developing cancer (Wu et al. 2016); second, as stated by Hasin et al., ‘given that most common, complex diseases develop over time and involve both environmental and genetic factors, full mechanistic insight will require coordinated sets of several omics data at multiple time points, collected from many disease relevant tissues’ (Hasin et al. 2017); third, as stated by Prof. Alan Attie, 'There are many steps between casual gene and phenotype at the level of body weight and blood sugar. Each step is subject to genetic variation, which can weaken links between gene and phenotype' (Delude 2015); and fourth, most major diseases result from long-term, complex interactions among numerous genetic and environmental factors, which would significantly decrease the precision for genomics-based diagnosis of diseases (Delude 2015).

There is evidence indicating that phenomic approach is a novel and revolutionary approach for accelerating profoundly the discoveries of novel risk factors (Andrews et al. 2021; Nikpay and Mohammadzadeh 2020; Went et al. 2020; Yang et al. 2022; You et al. 2022), diagnostic methods (Rahman and Rahman 2019; Viganò et al. 2012; Xu et al. 2022; Zhang et al. 2021, 2022a, e), and therapeutic strategies (Delude 2015; Robinson 2012; Zhang et al. 2021). Compared to genomic approach for studying diseases, phenomic studies on diseases determine a number of cross-scale phenomic parameters on several levels, including molecular level, cellular level, tissue and organ level, and systemic level (Table 1).

**Table 1:** Representative technologies and measured phenomic parameters of human subjects in phenomic studies on diseases

Levels of phenomic parameters	Representative technologies and measured phenomic parameters of human subjects in phenomic studies on diseases
Molecular level	(1) Routine blood assays (Moravej Aleali et al. 2019)
	(2) Routine urine assays (Mishriki et al. 2013)
	(3) Routine stool assays (Kasırga 2019)
	(4) Genetic analyses (Gorcenco et al. 2020)
	(5) Epigenetic assays (Heyn and Esteller 2012)
	(6) Proteomic assays (D’Alessandro and Zolla 2017)
	(7) Metabolomic assays (Nordström and Lewensohn 2010)
	(8) Assays on body fluids (Boukouris and Mathivanan 2015)
	(9) Tumor biomarker assays (Lianidou et al. 2015)
	(10) Cytokine assays (Ueland et al. 2015)
	(11) Blood gas analysis (Thorp and Rushing 1999)
Cellular level	(1) Routine blood assays (Moravej Aleali et al. 2019)
	(2) Flow cytometry (Monneret et al. 2019)
	(3) Single-cell analysis (Gohil et al. 2021)
	(4) Immunophenotyping (Dermawan et al. 2021)
Tissue and organ level	(1) Magnetic Resonance Imaging (MRI) (Choyke et al. 2003; González 2012; Muccilli et al. 2018)
	(2) Computerized Tomography (CT) (Furukawa et al. 2004; Zeman et al. 1995)
	(3) Positron Emission Tomography (PET) (Deri et al. 2013; Tian et al. 2004)
	(4) X-rays (Jaeger et al. 2014)
	(5) Endoscope (Tajiri et al. 2010)
	(6) Bronchoscope (Ishiwata et al. 2020)
	(7) Ultrasound (Deshpande et al. 2010; Jensen 2007)
	(8) Radionuclide scanning (Datz and Taylor 1985; Pattou et al. 1998)
	(9) Electrocardiogram (Rudy and Burnes 1999)
	(10) Electroencephalogram (Michel and Murray 2012)
	(11) Electromyogram (Tankisi et al. 2020)
	(12) Cervical biopsy (Drezek et al. 2003)
	(13) Determinations of psychology and mental health (Cheng et al. 2019)
Systemic level (Hellmann et al. 2018)	(1) Weight
	(2) Height
	(3) Sex
	(4) Body temperature
	(5) Heart rate
	(6) Blood pressure
	(7) Behavior
Other information (Hellmann et al. 2018)	(1) Age
	(2) Personal history of diseases
	(3) Family history of diseases
	(4) Occupation
	(5) Educational level
	(6) Marriage status
	(7) Status of smoking and drinking

The difference between conventional studies on diseases and phenomic studies on diseases may be analogous to the difference between western blot assays and proteomics. Based on scientific principles, it is highly reasonable to use the changes of human phenomes as the biomarkers for incidence, diagnosis and therapeutic effects of diseases: since human body is a system consisting of numerous closely associated components, theoretically, it is impossible that the pathological changes of a certain component of the system is a fully independent event. Instead, the pathological changes could be associated with the changes of certain other components in the system. Therefore, through comprehensive determinations and analyses of the phenomic information of human body, there should be significant potential to expose novel risk factors, diagnostic biomarkers and prognostic biomarkers.

## Major Scientific Questions and Technical Questions for Phenomic Studies on Diseases

The major scientific questions for phenomics-based studies on diseases include at least the following unanswered questions:

1. What are the phenomic maps of diseases?
2. What are the major differences between the phenomic maps of the patients of certain diseases and the phenomic map of healthy subjects?
3. What are the major differences among the phenomic maps of the patients of various diseases?
4. What are the phenomic parameters that are key risk factors for diseases? What are the genes and proteins that are closely associated with the risk factors?
5. What are the phenomic parameters that can be used as novel diagnostic biomarkers of diseases? What are the genes and proteins that are closely associated with the new diagnostic biomarkers?
6. What are the phenomic parameters that can be used as novel prognostic biomarkers for diseases? How to establish protocols and models of precision therapies for diseases on the basis of phenomic information?

The following unanswered questions belong to the major technical questions for phenomic studies on diseases:

1. What are the standards for the measurements and data analyses in phenomic studies on diseases?
2. What are the novel and valuable phenomic parameters that should be measured in phenomic studies on diseases? How to develop novel methods and equipment for phenomic studies on diseases under clinical settings?

## Significance and Theoretical Basis for Phenomics-Based Studies on Novel Diagnostic Biomarkers for Major Diseases

Early, non-invasive and precise diagnosis of major diseases is of pivotal significance for effective treatment of the diseases. However, there has been significant deficiency in this type of diagnostic biomarkers and methods, which has severely limited the therapeutic efficacy of the diseases. There have been numerous findings indicating that certain phenomic parameters of human body can become valuable biomarkers for diagnosis: first, the pathological changes of eye’s micro-vessels and the fiber layer of retina's nerves can become the biomarkers for diagnosis of early-stage Alzheimer’s disease (Berisha et al. 2007; Lu et al. 2010; Patton et al. 2005). Second, there have been studies indicating that the autofluorescence (AF) of the skin and the eyes can become diagnostic biomarkers for diabetes (Sparrow et al. 1992), which originates from the advanced glycation products (AGEs) of collagen (Bos et al. 2011; Mulder et al. 2006). The increased AF can also indicate the complications of diabetes such as micro-vessel damage (Gerrits et al. 2008) and kidney failure (Mulder et al. 2006). Third, our recent study has indicated that decreased green AF of lung parenchyma is a biomarker of lung cancer tissues (Zhang et al. 2022d); our latest study has indicated that the green AF of the Index Fingernails is a novel biomarker for non-invasive, rapid and economic determinations on the status of tobacco smoking (Zhang et al. 2022b); and our study has also indicated that ultraviolet (UV) radiation-induced skin's green AF is a biomarker for both non-invasive prediction of UV-induced skin damage and non-invasive evaluations of the dosages of UV exposures of the skin (Zhang and Ying 2022). Fourth, several reports have indicated that the pathological changes of the nails may become diagnostic biomarkers for diseases: the patients of Systemic Lupus Erythematosus have significant morphological changes in their nail's micro-vessels (Ciołkiewicz et al. 2010); pathological changes of the micro-vessels for nail fold microcirculation are a diagnostic biomarker for Systemic Sclerosis (Koenig et al. 2008; Hoogen et al. 2013); and the levels of Cd, Mn, Ni and Pb in the nails of prostate cancer patients are significantly higher than those of healthy subjects (Qayyum and Shah 2014). Fifth, the porphyrin's AF in the blood can become biomarkers for both cancer diagnosis (Masilamani et al. 2004) and differentiation between benign and malignant tumors (Koenig et al. 1996). Sixth, multiple studies have indicated that the pathological changes of the hair may become diagnostic biomarkers for multiple diseases (D’Anna-Hernandez et al. 2011; Denny et al. 2013; Price et al. 1980; Russell et al. 2012; Yamada et al. 2007).

During the last several years, our study has indicated that epidermal green AF can become novel biomarkers for non-invasive, label-free, economic and rapid evaluations of the levels of inflammation and oxidative stress in the body, which is generated from keratin 1 and/or keratin 10 in the keratinocytes at the spinous layer of the epidermis (Zhang et al. 2020). We have also found significant differences between the green AF intensity of healthy subjects and that of AIS patients (Wu et al. 2018b), lung cancer (Zhang et al. 2022a), and Parkinson’s disease (Wu et al. 2018a) at multiple skin’s locations examined. The AF properties have shown excellent promise for becoming new diagnostic biomarkers for the diseases, e.g., solely based on the skin's green AF intensity at the Dorsal Index Fingers of AIS patients, the area under curve (AUC) for differentiating AIS patients from the healthy subjects is 0.88 (Wu et al. 2018b). Based on these findings, we have proposed a novel theory for diagnosis of diseases—‘Pattern of AF Theory’: ‘Several properties of the keratin-based green AF at multiple locations of the skin, including AF intensity, AF asymmetry, locations of AF increase, and structure of the AF images, form the ‘Pattern of AF’. Each major disease has its characteristic ‘Pattern of AF’, based on which major diseases may be diagnosed (Wu et al. 2018b)’.

**Fig. 1:**
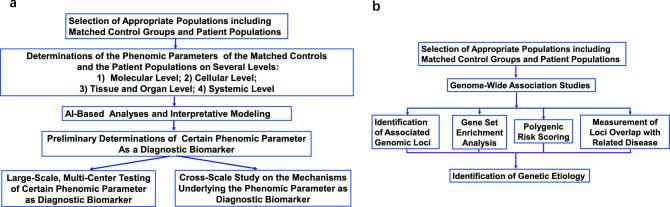
**a** A proposed diagram for phenomic studies on diagnostic biomarkers of diseases. **b** A diagram for genetic studies on diagnostic biomarkers of diseases

It is expected that phenomic studies on diagnostic biomarkers may profoundly enhance our capacity to discover novel diagnostic biomarkers and methods. Compared with conventional approaches, phenomics-based studies on diagnostic biomarkers have the following distinct merits: first, in the phenomics-based studies, the associations between the disease incidence and numerous phenomic parameters can be analyzed by AI, which may enhance greatly the efficiency for discovering new diagnostic biomarkers; second, the big data from phenomics-based studies enable us to elucidate the relationships among the diagnostic biomarkers and various phenomic parameters of patients; and third, the phenomics-based studies do not require findings from previous animal studies or cell culture studies. A proposed diagram for phenomic studies on diagnostic biomarkers of diseases is shown as Fig. 1a. For comparisons, a diagram for genetic studies on diagnostic biomarkers of diseases is shown as Fig. 1b.

## Significance and Theoretical Basis for Phenomic Studies on the Risk Factors of Major Diseases

Disease prevention is a crucial strategy for overcoming the challenges from major diseases. Discovery of risk factors is required for not only prevention of major diseases, but also establishment of novel strategies for elucidating the mechanisms underlying the pathogenesis of the diseases. Conventional approaches for discovering risk factors of diseases have three major limitations: first, these studies usually focus on a single hypothesis or a single factor, which leads to relatively low efficiency in discovering new risk factors; second, the data from these studies are usually not rich enough to expose the relationships between the risk factor and other biological parameters of the subjects; and third, previous findings from biological experiments are usually required for the design of these studies.

**Fig. 2:**
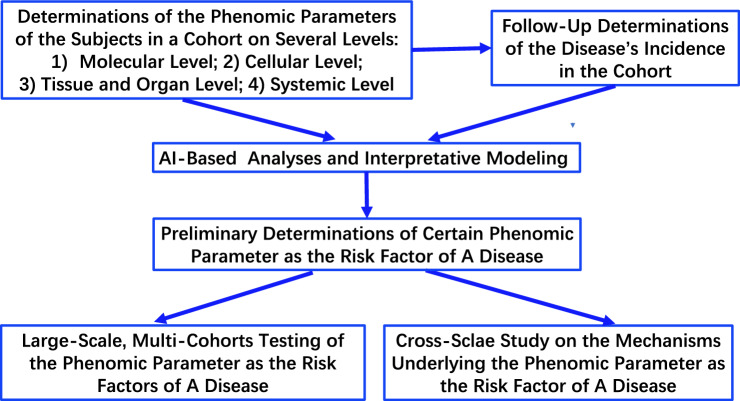
A proposed diagram for phenomic studies on risk factors of diseases

Applications of phenomics may enhance revolutionarily our capacity to discover new risk factors of diseases, which have the following distinct merits compared to conventional approaches: first, phenomics-based studies can search for multiple new risk factors in a single study by investigating the associations between the disease incidence and a number of phenomic parameters of the subjects (Andrews et al. 2021; Nikpay and Mohammadzadeh 2020; Went et al. 2020; Yang et al. 2022; You et al. 2022); second, the rich and multi-dimensional data from the phenomics-based studies enable us to investigate the relationships between the risk factors and other phenomic parameters of the subjects, which can profoundly enhance our understanding on the mechanisms underlying the associations between the risk factors and disease incidence; and third, the phenomics-based studies are big data-driven studies that do not require the findings from previous biological experiments. A proposed diagram for phenomic studies on risk factors of diseases is shown as Fig. 2.

## Significance and Theoretical Basis for Phenomic Studies on Precision Therapies for Major Diseases

A number of clinical studies have found that under many circumstances, a specific therapy is effective in treating only one specific group of the patients of a certain disease (Jones and Baldwin 2018). Therefore, it is critical to establish personalized therapeutical protocols so as to conduct precision therapies. However, there has been significant deficiency in the strategies and methods for developing precision therapies. Genetic analysis has been a major strategy for establishing personalized therapies, which has shown significant limitations due to both complexity of diseases and significant impact of environmental factors on the development of diseases (Delude 2015; Hennekam and Biesecker 2012; Robinson 2012).

**Fig. 3:**
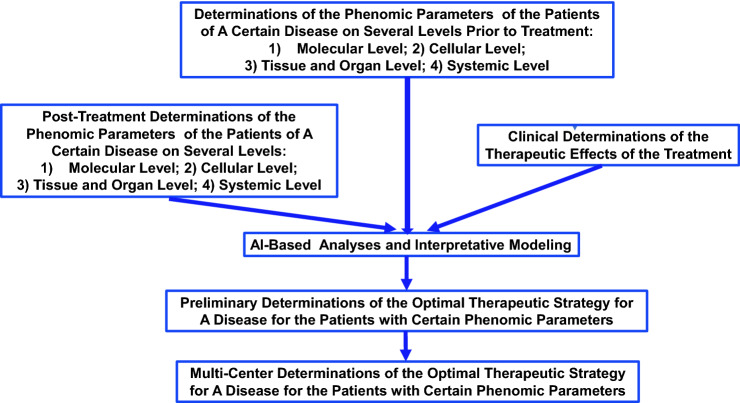
A proposed diagram for phenomic studies on precision therapies of diseases

Phenomics appears to be one of the key areas for future development of precision medicine (Denny and Collins 2021). It is expected that by combining phenomic studies and genomic studies, the protocols of precision therapies may be established: Through AI-based investigation of the massive, multi-dimensional phenomic data collected before, during and after a certain therapy, there is a significant possibility to discover that certain phenomic parameters of patients are strongly associated with excellent, satisfactory or poor clinical outcomes. Based on these studies, a certain therapy may be applied only for the patients who have certain characteristic phenomic parameters and genomic properties, which may lead to establishment of precision therapies for major diseases. A proposed diagram for phenomic studies on precision therapies is shown as Fig. 3.

## Phenomic Studies on Diseases may Significantly Improve both Western Medicine and Traditional Chinese Medicine

Phenomic studies on diseases may profoundly transform both Western medicine and traditional Chinese medicine. One distinct property of Western medicine is that under most circumstances, only the key disease-related organ is the major target for medical examinations, which largely ignores the significance of the other phenomic changes of the patients. Phenomic studies on diseases may profoundly enhance the capacity of Western medicine by examining not only the key disease-related organ, but also other phenomic changes of the patients, which would significantly enhance the possibility of discovering new diagnostic biomarkers and establishing precision therapies.

Compared to Western medicine, traditional Chinese medicine relies highly on the observations of phenomic changes of the body as a system. However, traditional Chinese medicine lacks both precise measurements on the biological properties of the body and precise data-based elucidation of the theories of traditional Chinese medicine. It is expected that phenomic studies on diseases may provide an important basis for systemic and quantifiable explanations for the theories of traditional Chinese medicine.

## Potential of Phenomic Studies for Development of Medical Instrumentation

The development of medical instrumentation such as medical imaging equipment and medical robotics has played an important role in the advancement of modern medicine, e.g., Magnetic Resonance Imaging (MRI) and Computerized Tomography (CT) imaging plays pivotal roles in AIS diagnosis (El-Koussy et al. 2014; Vilela and Rowley 2017); and Positron Emission Tomography (PET) imaging plays a critical role in cancer diagnosis (Zhang et al. 2022e). However, due to such factors as the cost of the medical instrumentation, there is significant deficiency in the medical equipment in many areas around the world, particularly in rural areas (Hricak et al. 2021; Khatib et al. 2018). Even in developed countries, MRI imaging resource is deficient in acute settings (Wintermark et al. 2013). This deficiency has severely limited the quality and efficiency of diagnosis and treatment of major diseases such as AIS. Phenomic studies on diseases have established a novel pathway for determining the association between incidence of a disease and a certain phenomic parameter that is usually overlooked in previous studies. Novel, economic and efficient diagnostic equipment may be established, based on phenomics-based discovery of novel diagnostic biomarkers, for example, a Portable Human Fluorescence Imaging Device has been invented in recent years for diagnosis of such diseases as AIS and lung cancer (Wu et al. 2018b; Zhang et al. 2022a).

## Significance and Theoretical Basis for Phenomic Studies on Human Wellness

In addition to its wide applications to the studies on diseases, phenomics has extensive applications on studies on human wellness. One example for this type of study is phenomic study on human aging: human aging is highly complex and systemic alterations of the biological properties of human body, which can be affected by multiple mechanisms (Campisi et al. 2019). To obtain comprehensive picture of aging so as to elucidate the essential mechanisms of aging and discover hallmarks of aging, it is critical to conduct comprehensive determinations on the multi-dimensional and cross-scale phenomic parameters of both young and aging subjects. Rapid increases in applications of Omics approaches and AI in aging studies have established a strong basis for phenomics-based studies on aging (Mkrtchyan et al. 2020). It is expected that phenomic studies on aging can not only provide remarkably more comprehensive information regarding the biological alterations in the aging process, but also enable us to elucidate the mechanisms of aging by conducting AI-based, cross-scale determinations of the relationships among the microscopic and macroscopic properties of human body in the aging process.

## Significance of Medical Imaging in Phenomic Studies on Diseases

Medical imaging, including MRI, CT and PET, has become indispensable tools for diagnosis of numerous diseases such as stroke (El-Koussy et al. 2014; Vilela and Rowley 2017) and cancer (Zhang et al. 2022e). As stated below, cumulating evidence has indicated that medical imaging techniques will play a critical role in phenomic studies on diseases, which is established on the basis of rapid technical development of medical imaging technologies, increased integration between medical imaging technologies and multi-omics technologies, and accelerated applications of AI technology in medical imaging.

AI technology has played an increasingly important role in MRI image analyses (Sheth and Giger 2020). Cumulating evidence has also indicated the promise that MRI datasets can be integrated with Omics datasets (Huang and Shih 2014; Leithner et al. 2018; Li et al. 2021b). To integrate MRI images with omics datasets, imaging techniques should be fast, accurate, safe and convenient. As a critical technical advance of MRI, the speed of MRI equipment has been significantly increased by the development of parallel computing technology and Dynamic Acceleration technology (Hamilton et al. 2017; Huang and Shih 2014). Collectively, these major advances of MRI technique have established strong basis for MRI to become a key technology in phenomic studies on diseases.

AI technology has played an increasingly important role in CT image analyses (Shi et al. 2021). Cumulative evidence has also indicated the promise that CT datasets can be integrated with Omics datasets (Huang and Shih 2014; Leithner et al. 2018; Li et al. 2021b). As a major technical advance of CT, radiation dosages of CT have been significantly reduced by several technologies (Huang and Shih 2014). Collectively, these major advances of CT technique have established solid basis for CT to become an important technology in phenomics-based studies on diseases.

PET is a major molecular imaging technology that can provide non-invasive whole-body phenotyping, which plays critical roles in diagnosis of such major diseases as cancer (Jin et al. 2022; Zhang et al. 2022e) and Alzheimer’s disease (Chapleau et al. 2022; Cook et al. 2014). PET is capable of assessing precisely multiple biological processes in the body, including signal transduction, protein expression, receptor availability, transporter systems, and gene mutation (Zhang et al. 2022e). The major current advances of PET technology have enabled PET to play significantly greater role in phenomic studies on diseases: first, the key deficiency of PET imaging is its relatively low spatial resolution (Surti 2015; Wollenweber et al. 2016). To overcome this shortcoming, PET imaging has been integrated with CT imaging or MRI imaging to form hybrid imaging equipment, including PET/CT and PET/MRI (Tian et al. 2021). The hybrid imaging equipment can achieve the distinct technical merits of both imaging techniques: it can obtain patients' medical images in both microscopic and macroscopic scale in the same platform, which can indicate the pathological state of patients in molecular, structural and functional aspects (Tian et al. 2021); second, novel PET tracers other than β-2-[^18^F]-Fluoro-2-deoxy-D-glucose (^18^F-FDG) are expected to further enhance its capacity to decode phenotypes of diseases (Zhang et al. 2022e); and third, there have been rapid increases in the applications of AI in PET studies (Arabi et al. 2021; Sitek et al. 2021).

Due to the rapid progresses of medical imaging technologies, Tian et al. has proposed a novel concept for the emerging new pathology, named ‘Transpathology’ (Tian et al. 2021). There are three major concepts in ‘Transpathology’, including trans-scale, transparency, and translation (Tian et al. 2021): a trans-scale imaging mode is applied for evaluations of the pathological changes of patients; multimodal probe technology can make the whole body of patients ‘transparent’, leading to markedly increased understanding on the pathological changes of patients; and translation of the discoveries from basic research to clinical practice would accelerate the realization of precision medicine. 'With the development of genomics, proteomics, and metabolomics, the organic combination of macro and micro imaging technology, and the progress in information and AI technology, clinical pathology will be promoted toward the pattern of cross-scale, multi-mode 'transparent pathology' (Tian et al. 2021)'.

## Current Status of Phenomic Studies on Diseases

Twenty-seven clinical research centers from four countries including US and Canada established a joint research team in 2007 to initiate the Epilepsy Phenome/Genome Project (EPGP) (Abou-Khalil et al. 2013). They have established detailed procedures for cohort study and conducted determinations of the variety of phenomes (Abou-Khalil et al. 2013). Their study has indicated that the white people in the Caucasian region have high probability of developing Generalized Epilepsy compared with other races (Friedman and Fahlstrom 2013). They have also found that cerebellar gyrus can be used as a phenotype for predicting and examining the incidence of epilepsy (Shain et al. 2013). An Endometriosis Phenome and Biobanking Harmonisation Project was established, which applies both phenomic and genomic approaches to study endometriosis (Zondervan et al. 2016). The scientific goal of this project is to enhance classifications of endometriosis on the basis of both phenomic and genomic information of the patients (Zondervan et al. 2016).

A research team from Denmark proposed that based on the analyses on human protein complex, virtual prediction of familial diseases can be conducted (Lage et al. 2007). It was also reported that by applying machine learning technology to study Autism spectrum disorder, thousands of phenotypes that indicate cognitive ability, movement capacity and biorhythm can be analyzed (Bruining et al. 2014). Multiple phenomic studies on neurological diseases using animal models have been reported, e.g., it was reported that by machine learning-based analyses on numerous phenotypes that indicate cognitive ability and movement capacity in an animal model of Huntington disease, the number of CAG repeats and the age of mice could be predicted precisely (Alexandrov et al. 2016).

There have also been certain progresses on the methods for analyzing phenomic data. Based on machine learning, Genome-wide association study (GWAS) and phenome-wide association study (PheWAS) have been used jointly to study the relationships between the genotypes and the phenotypes of Type II diabetes (Zheng et al. 2017). As a tool for analyzing the phenomic data of cardiovascular diseases, PheWAS has been developed to discover new diagnostic and prognostic biomarkers (Han et al. 2015). PheWAS has also been used to analyze the data from epidemiological studies, clinical trials and electronic medical records (Pendergrass and Ritchie 2015; Roden 2017).

The development of the China-based International Human Phenome Project, led by Prof. Li Jin, has provided a major driving force for the progress of phenomic research. One distinct progress of phenomic studies has also originated from the founding of the first international journal that focuses at studies on human phenomes –*Phenomics* (Jin 2021).

A recent study has applied the strategies and methods of phenomic study to investigate the underlying genetic basis for differences in facial morphology in East Asian and European populations (Zhang et al. 2022c): they were the first group to apply Polygenic Shape Analysis (PSA) to conduct simulation of facial morphology; their PheWAS analysis showed that the facial morphology-influencing loci also influence other parameters of human phenome; and they also discovered that the locus rs6843082 is strongly associated with the risk of developing atrial fibrillation. Their findings have implicated the associations between facial morphology and development of cardiovascular and cerebrovascular diseases.

One recent phenomic study has indicated a key role of limb development genes in influencing the outcome of fingerprint patterning (Li et al. 2022): by applying genome-wide scans of Han Chinese cohorts, the authors identified 18 loci that are associated with the fingerprint patterns across the digits; they established a role of *ecotropic viral integration site 1* (*EVI1*) in dermotoglyph patterning in mice; they found that *EVI1* plays a role in shaping the digits and limbs; 43 fingerprints-associated loci were identified by transethnic meta-analysis, with nearby genes being enriched for limb development pathways; and hand proportions were genetically correlated with fingerprint patterns. In general, the study has found a strong association between fingerprint patterns and the phenotypes of limbs, the mechanism underlying which is the finding that *EVI1* is the common gene that influences development of both limbs and fingerprint patterns.

A study by Li et al. reported their findings on high‑altitude acclimatization in a Chinese Han longitudinal cohort (Li et al. 2021a): they defined ‘composite phenotypes’ by developing a strategy that combines partial least squares path modeling and spectral clustering, which can be used to reveal hidden population's physiological heterogeneity in high-altitude acclimatization. In the evaluations of model fitness performance, a model based on composite phenotypes fit better than single trait-based model in modeling oxygen saturation changes in high-altitude acclimatization. The authors proposed that the new composite phenotype-based strategy may become a general strategy for studies on complex traits such as analyses of phenomics.

It has also been reported that cancer can be detected up to four years before current standard of care (Chen et al. 2020): a non-invasive blood test based on circulating tumor DNA methylation detected five common types of cancer in 88% of post-diagnosis patients with a specificity of 96%. Another study has also shown that higher serum levels of glutamate, tyrosine, acetate, glycine, and phenylalanine were negatively related to incidence of dementia, while higher serum levels of glutamine and O-acetyl glycoproteins were associated with increased risk of dementia (Cui et al. 2020).

Based on their PheWAS analysis, a recent study by Liu et al. has discovered novel links between genetically determined levels of liver enzymes and disease phenotypes (Liu et al. 2022): genetically determined alanine aminotransferase (ALT) levels were associated with liver-related diseases; genetically predicted alkaline phosphatase (ALP) levels were associated with pulmonary heart disease, hypercholesterolemia, phlebitis, and thrombophlebitis of lower extremities; genetically determined λ-glutamyl transferase (GGT) levels were associated with such diseases as chronic non-alcoholic liver disease and cholelithiasis; and genetically determined aspartate aminotransferase (AST) levels were associated with the diseases of a wide range of phenotypic categories.

By studying the Biobank cohort, the latest study by Yang et al. examined systematically the women-specific trajectory of the disease network, including blood and urine biomarkers as well as the role of baseline physical examination indexes (Yang et al. 2022): among 301 diseases, 82 diseases in women had odds ratios (ORs) > 1.2 and *p* < 0.00017 when compared to men, mainly including diseases in the endocrine, digestive and skeletal systems. Diseases with the highest ORs included breast diseases, hyperthyroidism, osteoporosis, and deformity of the toes.

The review of Nicolson stated the importance of molecular phenomics in deconvolving the systemic effects of Severe Acute Respiratory Syndrome Coronavirus 2 (SARS-CoV-2) infection and post-acute Corona Virus Disease 2019 (COVID-19) syndrome (Nicholson 2021): molecular phenomics mainly studies the chemical and biochemical signatures of cells and biofluids, as well as the characteristic changes of these signatures during the onset, development, and recovery phase of the disease. It is critical to obtain these pieces of information so as to provide molecular characterization of COVID-19 and elucidate the systemic effects of the disease.

## Major Challenges and Tasks Facing Phenomic Studies on Diseases

The major challenges and tasks facing phenomic studies on diseases include:

1. Since phenomic studies on diseases can generate multi-dimensional big data in the size that is remarkably larger than that obtained by conventional studies on diseases, effective analyses of the data are essential for establishing valuable models. While there has been certain progress on this research direction (Han et al. 2015; Yang et al. 2017), the methods for the data analyses are still deficient. It is critical to establish the strategies and methods for analyzing phenomic data.
2. It is necessary to establish standardized protocols and methods for the data acquirement, data storage, data distribution and data usages in the phenomic studies on diseases, which is required for effective analyses, sharing and development of unified phenomic data banks on the basis of the phenomic data obtained by various organizations.
3. It is necessary to discover novel phenomic parameters that should be included as the fundamental parameters for phenomic studies on diseases.
4. Numerous analytic equipment for measuring biological parameters has been designed mainly for laboratory use. It is necessary to develop equipment that is used for measurements of phenomic parameters of patients under clinical settings.
5. It is essential to realize the potential of phenomic data by effective data analyses and data sharing, so as to promote the establishment of novel technology and equipment for enhancing our capacity of diagnosis and treatment of patients.
6. It is necessary to conduct persistent searches for the developmental strategies for phenomic studies on diseases. It is also important to conduct studies on the ethical and regulatory guidelines for phenomics-based studies on diseases.
7. It is important to establish large-scale phenomic research centers that can conduct multidisciplinary research. The centers also hold the responsibility of education and training of talented people for this research field.
8. It is critical to conduct international cooperation, in which each country can use its unique strength and potential to promote the development of this important field.

## Conclusions

Systemic phenomics-based studies on diseases are critically needed, due to the profound potential of the studies. It is expected that phenomics will profoundly enhance human being's capacity in prevention, diagnosis and treatment of diseases. The multi-dimensional and muti-layer biomedical big data from phenomic studies on diseases would be an invaluable resource for both understanding the mechanisms of diseases and development of new biomedical technology and devices. It is expected that phenomics-based studies on diseases would become a key strategy for realizing precision medicine. With the development of the International Human Phenome Project, it is reasonable to expect that the ‘Golden Age of Phenomics’ is arriving.

## Data Availability

Not applicable.
